# Mechanism of the Treatment of Irritable Bowel Syndrome with Sini Powder and Tong Xie Yao Fang Decoction Based on Network Pharmacology

**DOI:** 10.1155/2022/3598856

**Published:** 2022-04-01

**Authors:** Rong Tang, Xiaoqing Peng, Xiaohong Zhou, Zhimin Zheng, Jiayu Yin, Hong Liu

**Affiliations:** ^1^Department of Pharmacy, Guangzhou First People's Hospital, School of Medicine, South China University of Technology, Panfu Road 1, Guangzhou 510180, Guangdong, China; ^2^Department of Traditional Chinese Medicine, The First Affiliated Hospital of Guangdong Pharmaceutical University, Gonghexiheng Street 1, Guangzhou 510080, Guangdong, China

## Abstract

This study used a network pharmacology approach to investigate the potential active ingredients of Sini Powder and Tong xie yao fang decoction and the underlying mechanisms in irritable bowel syndrome (IBS) treatment. The potential active ingredients of Sini Powder and Tong xie yao fang decoction were obtained from TCMSP databases, and the potential targets of the active ingredients were predicted and analyzed by using the Swiss Target Prediction database. T Genecard, DisGeNET, and OMIM databases were processed to screen the potential therapeutic targets in IBS. The interaction of overlapped candidates between the potential biotarget of herb extracts and the potential therapeutic target of IBS were analyzed by STRING website and visualized by the Cytoscape V3.8.0 software. Gene ontology (GO) analysis and Kyoto Genomics and Genomics Encyclopedia (KEGG) pathway were processed to categorize and map the potential biofunctions and effects of these candidates by using David database. *Result*. There were 139 predicted active components and 248 related biotargets of Sini Powder and Tong xie yao fang decoction which were involved in IBS treatment, and 522 annotations and 101 related pathways are obtained by enrichment analysis (*P* < 0.01, FDR < 0.05). The underlying mechanisms of Sini Powder and Tong xie yao fang decoction may be related to neuroactive ligand-receptor interaction, calcium, cAMP, and HIF-1 signaling pathways. In conclusion, our results showed that the effect and mechanism of Sini Powder and Tong xie yao fang decoction in IBS treatment were in multi-ingredient, multitargets and multipathways, which would provide several potential and promising strategies for the further research and development of Sini Powder and Tong xie yao fang decoction on IBS treatment.

## 1. Introduction

Irritable bowel syndrome (IBS) is a dysfunctional gastrointestinal disease, which is characterized by recurrent abdominal pain, and associated with abnormal fecal form and/or abnormal frequency of defecation [1, 2]. The nationwide prevalence of Chinese IBS is 1.4%–11.5% in 2020 [3]. The high-risk factors of IBS at least including high fat, high bio-amine, and high carbohydrate diets, or intestinal infection [4, 5]. IBS patients usually suffered from anxiety, depression, and physical discomforts, which largely reduced their life quality [6]. Up to date, the pathophysiological mechanism of IBS remains as a complex context, accumulating evidence have been shown that visceral hypersensitivity, mental disorder, gut dysbiosis, gastrointestinal motility disorders, and intestinal low-grade inflammation contributed to dysfunction of gut-to-brain axis, which in turn played important roles in IBS initiation [7–11]. The therapeutic goals of IBS have so far been confined to ameliorate syndromes and improve patients' life quality. Clinically, IBS treatments mainly includes diet, lifestyle, medical, mental, and behavioral interventions, besides, individualized management is more important and required [3, 12].

Traditional Chinese medicine has showed promising advantages in IBS treatment [13, 14]. Traditional Chinese medicine theory holds that liver stagnation and spleen deficiency play important roles in pathogeneses of IBS [15]. The combination of Sini Powder and Tong xie yao fang decoction has proven effective and been reported fewer adverse reactions in IBS treatment [16, 17]. Sini powder is derived from the treatise on febrile diseases and is composed of Chinese thorowax root, Radix Paeoniae Alba, trifoliate orange (Fructus Aurantii Immaturus), and Chinese licorice (*Glycyrrhiza uralensis*). Tong xie yao fang decoction is derived from Danxi's Mastery of Medicine and is composed of *Rhizoma Atractylodis*, Radix Paeoniae Alba, Citri Reticulatae Pericarpium (Chenpi), and *Saposhnikovia divaricate* root. Both Sini powder and Tong xie yao fang decoction are based on the principle of reconciling the function of liver and spleen. The combination of two formulas can disperse the stagnated main and collateral channels in liver and spleen, which in turn to restore their normal function to ameliorate diarrhea, abdominal pain, and abdominal distension symptoms in IBS patients [16, 17]. However, the potential active ingredients of Sini powder and Tong xie yao fang decoction and the underlying mechanisms remain largely elusive.

In the present study, we explored the effective components, and then screened the hub targets and the relevant signaling pathways of Sini powder and Tong xie yao fang decoction based on network pharmacology analysis. Thus, our results would provide a theoretical reference for the study of its pharmacological mechanism and its clinical application.

## 2. Materials and Methods

### 2.1. Screening of Active Ingredients of Sini Powder and Tong xie yao fang Decoction and Target Prediction

The traditional Chinese medicine systems pharmacology database and analysis platform (TCMSP) (https://tcmspw.com/) was used to retrieve Chinese medicines (Chinese thorowax root, Radix Paeoniae Alba, trifoliate orange Fructus aurantii Immaturus), and Chinese licorice (*Glycyrrhiza uralensis*), *Rhizoma Atractylodis*, Citri Reticulatae Pericarpium (Chenpi), and *Saposhnikovia divaricata* root, which are components of Sini powder and Tong xie yao fang decoction. The active components of these Chinese medicines were collected and screened based on oral bioavailability (OB) ≥ 30% and drug-likeness (DL) ≥ 0.18. The SDFs (structure data files) of the molecular structure of potential active components were obtained through the PubChem database (https://pubchem.ncbi.nlm.nih.gov/), and their potential targets were predicted using the Swiss Target Prediction database (https://www.swisstargetprediction.ch/). Their potential targets were finally subjected to gene standardization through the UniProt database (https://www.UniProt.org/).

### 2.2. Prediction of IBS-Related Targets

Using “irritable bowel syndrome” as the keyword, searches, and screening were conducted using disease gene databases such as DrugBank (https://www.drugbank.ca/), DisGeNET (https://www.disgenet.org/), Genecards (https://www.genecards.org), OMIM (https://www.omim.org/), and TTD (https://db.idrblab.net/ttd/). The obtained target genes were introduced into the UniProt database for gene standardization.

### 2.3. Construction of the Active Ingredient-Target Network of the Drugs

The targets obtained in the steps described in “1.1” and “1.2” were imported into the Venny 2.1 website to create a Venn diagram, and the common targets of Sini powder and Tong xie yao fang decoction for IBS were obtained. Cytoscape 3.8.0 software was used to construct a drug active ingredient-potential therapeutic target network. The degree of node connectivity represents the number of edges connected to a certain point. The greater the value is, the greater the importance of the point in the topological structure. The degree of node connectivity was calculated using Cytoscape software, and the important effective ingredients and targets of Sini powder and Tong xie yao fang decoction in the treatment of IBS were clarified.

### 2.4. Construction of the Protein Interaction Network (PPI)

The common targets obtained with the procedure described in “1.3” were imported into the STRING database (https://string-db.org/), the species “homo sapiens” was selected, and the threshold was set to >0.7. Free targets were hidden to obtain PPI networks. The data were imported into Cytoscape 3.8.0 software for visualization, and the core targets in the PPI network were obtained. The Molecular Complex Detection (MCODE) plug-in in Cytoscape 3.8.0 software was used to perform a modular analysis of the core targets of the PPI network. Degree cutoff = 2, degree cutoff > 2, node score cutoff = 0.2, K-Core = 2, and Max.Depth = 100 were set, and the features of the targets were analyzed to select core target modules.

### 2.5. Functional Annotation of Common Target Genes Using Gene Ontology (GO) and Kyoto Encyclopedia of Genes and Genomes (KEGG) Pathway Enrichment Analysis

The common targets obtained in step “1.3” were imported into the DAVID 6.8 database (https://david.ncifcrf.gov/) for GO and KEGG analysis. The screening conditions were set as *P* < 0.01 and FDR < 0.05. The top 20 enriched results were displayed, the results were visualized using the Image GP tool, and the analysis results were displayed in the form of bubble graphs.

## 3. Results

### 3.1. Screening of Active Ingredients of Sini Powder and Tong Xie and Target Prediction

According to the OB and DL parameters and after excluding the compounds that were not included in PubChem or for which the relevant target could not be predicted by the Swiss Target, the following active ingredients were screened from the TCMSP database: 14 Chinese thorowax root (including 3 duplicate compounds), 11 Radix Paeoniae Alba (including 4 duplicate compounds), 20 Fructus aurantii Immaturus (including 4 duplicate compounds), 87 *Chinese licorice* (including 6 duplicate compounds), 3 *Rhizoma Atractylodis*, 14 *Divaricate Saposhnikovia* root (including 3 duplicate compounds), and 4 Citri Reticulatae Pericarpium (including 3 duplicate compounds). A total of 139 active ingredients were finally obtained (Table 1). Chinese thorowax root, Radix Paeoniae Alba, Fructus aurantii Immaturus, Chinese licorice, *Rhizoma Atractylodis*, *Divaricate Saposhnikovia* root, and Citri Reticulatae Pericarpium are referred to as BR, PRA, AFI, GRER, AMR, SR, and CRP, respectively. The abovementioned 139 active ingredients were retrieved from the PubChem website and the Swiss Target Prediction database to obtain 9,846 targets. After screening and the removal of duplications, a total of 960 targets were obtained.

### 3.2. Construction of the Active Ingredient-Target Network of the Drugs

**Figure 1:**
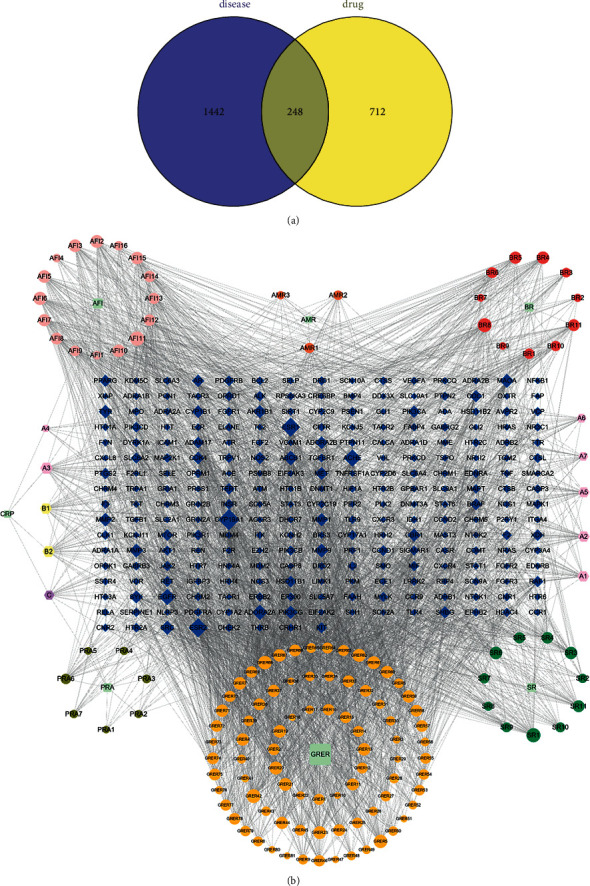
Active ingredient-target network of the drugs. (a) Venn diagram of related targets of active ingredients of Sini powder and Tong xie yao fang decoction in the treatment of irritable bowel syndrome. (b) Drugs-active components-target network diagram of Sini powder and Tong xie yao fang decoction in the treatment of irritable bowel syndrome.

Five disease databases—DrugBank, DisGeNET, Genecards, OMIM, and TTD—were searched, and 57, 429, 768, 623, and 27 of target sites were obtained, respectively. After summarizing the abovementioned targets and removing duplicate targets, 1,690 disease targets were finally obtained. A total of 248 common targets were obtained by taking the intersection of the targets obtained in the steps described in sections “2.1” and “2.2” (Figure 1(a)). Cytoscape 3.8.0 software was used to construct a network diagram of traditional Chinese medicine-active ingredient-potential therapeutic targets (Figure 1(b)). The diagram contains a total of 394 nodes and 3,313 edges. The degree of potential therapeutic targets of IBS (i.e., the number of compounds involved in the regulation of this target) was calculated. The top 10 potential targets were cytochrome P450 family 19 subfamily A member 1 (CYP19A1), estrogen receptor beta (ESR2), ESR1, acetylcholinesterase (ACHE), epidermal growth factor receptor (EGFR), adenosine A2A receptor gene (ADORA2A), ATP binding cassette subfamily B member 1 (ABCB1), v-src avian sarcoma (Schmidt-Ruppin A-2), viral oncogene homolog (SRC), monoamine oxidase A (MAOA), and matrix metalloproteinase 9 (MMP9). Additionally, a potential active compound can correspond to multiple potential therapeutic targets, such as obacunone, praeruptorin B, 7-acetoxy-2-methyl isoflavone, alfalfa toxin, poncimarin, licochalcone B, wogonin, and quercetin. This reflects the characteristics of the network structure of a single drug with multiple targets and multiple drugs that have the same target in the active ingredient-target network diagram, indicating that Sini powder and Tong xie yao fang decoction use a multitarget, multipathway, and multistep approach to treat IBS.

### 3.3. Construction of the PPI Network between the Active Ingredients of Sini Powder and Tong Xie Yao Fang Decoction and IBS

**Figure 2:**
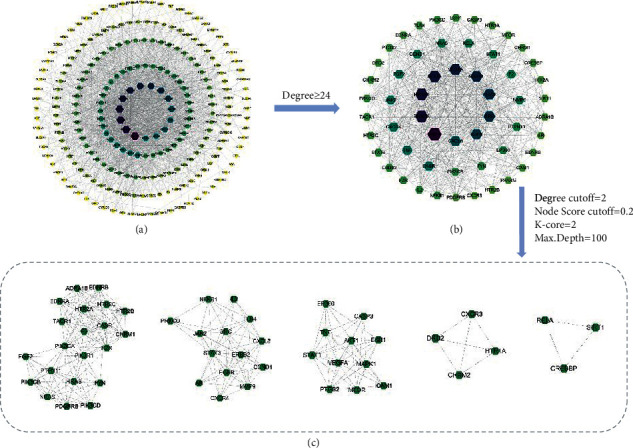
PPI networks of Sini powder and Tong xie yao fang decoction in the treatment of irritable bowel syndrome. (a) Bio-targets of Sini powder and Tong xie yao fang decoction in irritable bowel syndrome treatment. (b) Key biotargets from (a). (c) Modularization analysis of (b).

The 248 common targets obtained in the process described in “2.3” were imported into the STRING database to construct the PPI network. After the TSV file was obtained, it was imported into Cytoscape 3.8.0 software for visualization and network topology analysis. The PPI network contained a total of 232 nodes (16 target proteins are not involved in the interaction), and there were 1884 edges. The larger the degree value is, the larger the size of the hexagonal area and the darker the color (Figure 2(a)). The median degree value was 12, and a degree value ≥24 was used as the screening condition to obtain the core target proteins of Sini powder and Tong xie yao fang decoction in the treatment of IBS (Figure 2(b)). Of these, AKT Serine/Threonine Kinase 1 (AKT1, degree = 76); phosphatidylinositol-4, 5-Bisphosphate 3-Kinase Catalytic Subunit Alpha (PIK3CA, degree = 67); phosphoinositide-3-kinase regulatory subunit 1 (PIK3R1, degree = 66); signal transducer and activator of transcription 3 (STAT3, degree = 64); vascular endothelial growth factor (VEGF, degree = 62); mitogen-activated protein kinase 1 (MAPK1, degree = 60); H-Ras Proto-Oncogene; GTPase (HRAS, degree = 57); SRC (degree = 56); EGFR (degree = 54); and C-X-C motif chemokine ligand 8 (CXCL8, degree = 50) were obtained. The larger degree value of the target point reflects the high density of nodes and their surrounding nodes, which play important roles in the network. Therefore, these targets may be keys in the treatment of IBS. The MCODE plug-in of Cytoscape software was used for modular analysis of the screened core target proteins, and their characteristics were analyzed to screen the core target modules (Figure 2(c)). Selected modules have higher information transmission efficiency, and a single target has stronger interactions with other nodes.

### 3.4. GO Functional Enrichment and KEGG Pathway Enrichment Analysis

**Figure 3:**
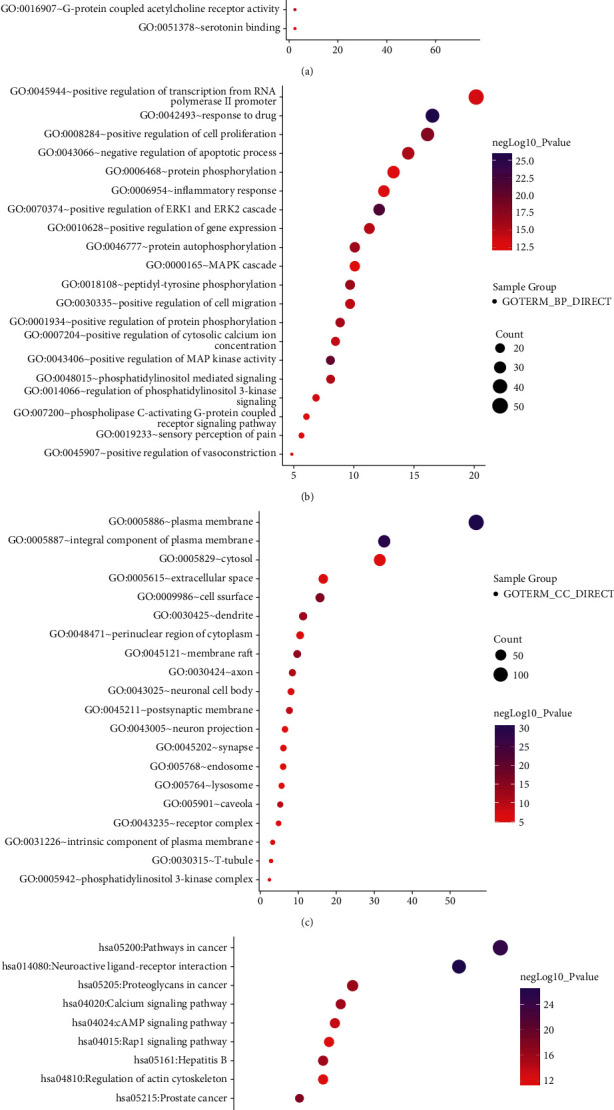
GO and KEGG PATHWAY enrichment analysis for targets of Sini powder and Tong xie yao fang decoction related to IBS. (a) Molecular function. (b) Biological processes. (c) Cellular component. (d) Bubble chart of KEGG pathway analysis. The order of importance was ranked by −log10 (*P* value) and gene number. In terms of *P* values, in ascending order, the top 20 GO functions and KEGG pathways were used to make bubble plots.

When *P* < 0.01 and FDR < 0.05, a total of 522 GO functions were enriched in common targets (including 79 molecular function items, 386 biological process items, and 57 cell component items). In terms of molecular functions, these functions were mainly enriched in protein binding, ATP binding, drug binding, enzyme binding, 5-hydroxytryptamine binding, protein kinase activity, protein serine/threonine kinase activity, protein tyrosine kinase activity, transcription factor binding, etc (Figure 3(a)). In terms of biological process, they were mainly enriched in the transcription of RNA polymerase II promoter, cell proliferation, gene expression, positive regulation of the ERK1 and ERK2 cascade, drug response, negative regulation of apoptosis process, protein phosphorylation, inflammatory response, and protein autophosphorylation, MAPK cascade, etc (Figure 3(b)). In terms of molecular components, they were mainly enriched in the plasma membrane, dendrites, synapses, and neuronal cell bodies (Figure 3(c)). The KEGG pathway enrichment screening resulted in 101 signaling pathways. The results suggest that cancer, neuroactive ligand-receptor interaction, proteoglycan in cancer, calcium ion signal, cAMP, hepatitis B, hypoxia-inducible factor 1-alpha (HIF-1), pancreatic cancer, and other signal pathways may be closely related to the pathogenesis of IBS (Figure 3(d)).

## 4. Discussion

Liver stagnation and spleen deficiency are important pathogenies of IBS in the theory of the traditional Chinese medicine. Sini powder and Tong xie yao fang decoction, which restrict the liver and support the spleen, have good indications and clinical efficacy for IBS [16, 17].

In the present study, the topological properties of the active ingredients-potential target network of Sini powder and Tong xie yao fang decoction were analyzed. We screened and picked up the ingredients with the highest degree value, such as obacunone, praeruptorin B, 7-acetoxy-2-methyl isoflavone, alfalfa toxin, poncimarin, licochalcone B, wogonin, and quercetin for the subsequent analysis. Obacunone is a natural compound that is widely present in plants in the Rutaceae family. It has anti-inflammatory, antitumor, antioxidative, and antipulmonary fibrosis effects. It has been confirmed in animal models that obacunone can inhibit the production of proinflammatory mediators [18]. It also alleviate colitis by improving the abnormal composition of intestinal microbiota and suppressing excessive activation of toll-like receptors (TLRs)/NF-*κ*B signaling cascades to [19]. Licochalcone B can exert anti-inflammatory effects by inhibiting the phosphorylation of nuclear factor kappa B (NF-*κ*B) P65 in the lipopolysaccharides (LPS) signaling pathway [20]. Wogonin is a pure natural flavonoid with a variety of biological activities, such as antiviral, anti-inflammatory, anticancer, neuroprotective, and antianxiety effects [21]. Previous studies have showed that wogonin can be used to treat colitis by inducing the expression of transcription factor HIF-1*α* through the protein kinase B/glycogen synthase kinase *β* (AKT/GSK3*β*) signaling pathway to increase interleukin 10 (IL-10) production [22]. Synergistic treatment using quercetin and 5-aminosalicylic acid improved symptoms and signs in a rat model of IBS after infection and improved the therapeutic effect [23]. As a coumarin-based component, praeruptorin B has been confirmed to have anti-inflammatory, antioxidant, antitumor, and analgesic activities [24, 25]. However, there are few reports on the treatment of IBS using praeruptorin B. In the present study, our results and the others suggest that the ingredients of the Sini powder and Tong xie yao fang decoction mainly served as anti-inflammatory mediators which indicate that the combined decoction might have a promising therapeutic value for the IBS treatment. Moreover, one potential valuable ingredient praeruptorin B was selected in the present study, which might be a promising target for IBS further research and drug discovery.

We next investigate the underlying mechanism of the valuable ingredients in the Sini powder and Tong xie yao fang decoction. Top ten core targets, such as AKT1, PIK3CA, PIK3R1, STAT3, VEGF, MAPK1, HRAS, SRC, EGFR, and CXCL8 were selected and further analysis. Previous studies have showed that overexpression of microRNA495 downregulated STAT3 to improve intestinal mucosal barrier function in ulcerative colitis [26]. Cell adhesion molecule 1 (CADM1) also would inhibit STAT3 signaling pathway to improve the intestinal barrier function of rats with IBS-D [27]. EGFR is a transmembrane receptor tyrosine kinase in the ErbB family that can promote intestinal development, regulate tight-binding protein expression, inhibit oxidative stress-induced apoptosis, and reduce intestinal epithelial colonization by inducing the autophosphorylation of RTK and the subsequent activation of the Ras/MAPK, PI3K/AKT, and PLC-*γ*/PKC signaling pathways, and it plays an active role in regulating intestinal permeability and promoting the integrity of the intestinal barrier [28]. VEGF is one of the most important factors in gastrointestinal mucosal remodeling, mucosal defense, and ulcer healing. The antiulcer drug sofalcone can prevent gastric mucosal injury through the upregulation of VEGF production mediated by the nuclear factor erythroid-2 related factor 2/hemeoxygenase-1 (Nrf2/HO-1) pathway [29]. Camellia oil can improve ketoprofen-induced gastrointestinal mucosal injury by upregulating HO-1 and VEGF [30]. The ERK/MAPK pathway can increase the expression level of intestinal tight junction proteins to enhance the intestinal barrier function in dogs with splenic asthenia syndrome [31]. Taken these together, the Sini powder and Tong xie yao fang decoction might improve intestinal barrier function, regulate intestinal permeability, exert a protective effect on the gastrointestinal mucosa, and inhibit the inflammatory response in IBS treatment.

Our results further showed that the top ten core genes mainly involved cell proliferation, inflammatory response, protein phosphorylation, ERK1/2 cascade, MAPK cascade, neurons, 5-hydroxytryptamine (5-HT) binding, and protein binding by GO functional enrichment analysis. 5-HT is a neurotransmitter in the intestinal tract that is considered an important signaling molecule. It mainly acts on various receptors on smooth muscle, enteric neurons, intestinal cells, and immune cells to regulate various intestinal functions. The symptoms of IBS (such as visceral sensitivity and gastrointestinal motility disturbance) are related to the level of 5-HT in the intestine. Previous studies have showed that an increase in 5-HT levels is correlated with diarrhea-dominant IBS. A decrease in the 5-HT level is correlated with constipation-dominant IBS [32]. The KEGG pathway enrichment analysis showed that the treatment of IBS with Sini Powder and Tong xie yao fang decoction involves multiple signaling pathways, such as cancer, neuroactive ligand-receptor interaction, calcium ion signaling, cAMP, HIF-1, and hepatitis B. The cAMP signaling pathway maintains the stability of the internal and external environment of intestinal cells, improves enteric nerve function, and regulates intestinal secretion and absorption [33]. PKA may mediate SP through cAMP signaling to regulate visceral sensitivity and enhance gastrointestinal motility [34]. In the rat model of diarrhea-dominant IBS, the expression levels of the L-type voltage-gated calcium channels Cav1.2 and Cav1.3 in the colon was found to be elevated to different degrees, suggesting that the increase in the L-type voltage-gated calcium channel may be the molecular basis of colonic motility disorders [35]. The T-type voltage-gated calcium channel Cav3.2 is upregulated in IBS and participates in visceral hypersensitivity, which is closely related to the occurrence and development of IBS [36]. HIF-1*α* is a sensitive indicator of intestinal hypoxia and is related to the maintenance of the intestinal barrier. Studies have confirmed that intestinal epithelial HIF-1*α* is an important protective factor against colitis and can effectively alleviate inflammatory colonic injury [37]. Taken these together, the Sini powder and Tong xie yao fang decoction might regulate intestinal nerve function, improve visceral sensitivity and gastrointestinal motility, and maintain intestinal barrier function to treat IBS by integrating these multi-pathways.

In summary, this study investigated the potential material basis and mechanism of action of the Sini powder and Tong xie yao fang decoction in the treatment of IBS through a network pharmacology method. The main mechanism of Sini powder and Tong xie yao fang decoction in the treatment of IBS may be the regulation of intestinal 5-HT levels to improve intestinal nerve function, act on calcium channels to improve visceral sensitivity, maintain intestinal barrier function to provide mucosal protection, and inhibit inflammatory responses. In general, the drug has multi-ingredient, multitarget, and multipathway characteristics and plays a therapeutic role through the synergistic effect of multipathway system regulation. The present study had provided an alternative potential strategy for IBS treatment. However, more evidence from the IBS animal model and IBS patients should be evaluated in the future studies.

## Figures and Tables

**Table 1:** List of active components of Sini powder and Tong xie yao fang decoction.

Herb	MOL ID	Molecule name	ID	OB%	DL
Radix bupleuri	MOL001645	Linoleyl acetate	BR1	42.1	0.2
Radix bupleuri	MOL002776	Baicalin	BR2	40.12	0.75
Radix bupleuri	MOL000449	Stigmasterol	BR3	43.83	0.76
Radix bupleuri	MOL000354	Isorhamnetin	A1	49.6	0.31
Radix bupleuri	MOL000422	Kaempferol	B1	41.88	0.24
Radix bupleuri	MOL004598	3, 5, 6, 7-tetramethoxy-2-(3, 4, 5-trimethoxyphenyl) chromone	BR4	31.97	0.59
Radix bupleuri	MOL004609	Areapillin	BR5	48.96	0.41
Radix bupleuri	MOL013187	Cubebin	BR6	57.13	0.64
Radix bupleuri	MOL004644	Sainfuran	BR7	79.91	0.23
Radix bupleuri	MOL004653	(+)-anomalin	BR8	46.06	0.66
Radix bupleuri	MOL004702	Saikosaponin c_qt	BR9	30.5	0.63
Radix bupleuri	MOL004718	*α*-spinasterol	BR10	42.98	0.76
Radix bupleuri	MOL000490	Petunidin	BR11	30.05	0.31
Radix bupleuri	MOL000098	Quercetin	A2	46.43	0.28
White peony root	MOL001918	Paeoniflorgenone	PRA1	87.59	0.37
White peony root	MOL001919	(3S, 5R, 8R, 9R, 10S, 14S)-3, 17-dihydroxy-4, 4, 8, 10, 14-pentamethyl-2, 3, 5, 6, 7, 9-hexahydro-1H-cyclopenta[a]phenanthrene-15, 16-dione	PRA2	43.56	0.53
White peony root	MOL001921	Lactiflorin	PRA3	49.12	0.8
White peony root	MOL001924	Paeoniflorin	PRA4	53.87	0.79
White peony root	MOL001925	Paeoniflorin_qt	PRA5	68.18	0.4
White peony root	MOL001928	Albiflorin_qt	PRA6	66.64	0.33
White peony root	MOL001930	Benzoyl paeoniflorin	PRA7	31.27	0.75
White peony root	MOL000211	Mairin	A7	55.38	0.78
White peony root	MOL000358	Beta-sitosterol	A6	36.91	0.75
White peony root	MOL000359	Sitosterol	C	36.91	0.75
White peony root	MOL000422	Kaempferol	B1	41.88	0.24
Fructus aurantii immaturus	MOL013276	Poncirin	AFI1	36.55	0.74
Fructus aurantii immaturus	MOL013277	Isosinensetin	AFI2	51.15	0.44
Fructus aurantii immaturus	MOL013279	5, 7, 4′-trimethylapigenin	AFI3	39.83	0.3
Fructus aurantii immaturus	MOL013428	Isosakuranetin-7-rutinoside	AFI4	41.24	0.72
Fructus aurantii immaturus	MOL013430	Prangenin	AFI5	43.6	0.29
Fructus aurantii immaturus	MOL013435	Poncimarin	AFI6	63.62	0.35
Fructus aurantii immaturus	MOL013436	Isoponcimarin	AFI7	63.28	0.31
Fructus aurantii immaturus	MOL013437	6-methoxy aurapten	AFI8	31.24	0.3
Fructus aurantii immaturus	MOL001798	Neohesperidin_qt	AFI9	71.17	0.27
Fructus aurantii immaturus	MOL001803	Sinensetin	AFI10	50.56	0.45
Fructus aurantii immaturus	MOL001941	Ammidin	A5	34.55	0.22
Fructus aurantii immaturus	MOL013352	Obacunone	AFI11	43.29	0.77
Fructus aurantii immaturus	MOL002914	Eriodyctiol (flavanone)	AFI12	41.35	0.24
Fructus aurantii immaturus	MOL004328	Naringenin	B2	59.29	0.21
Fructus aurantii immaturus	MOL005100	5, 7-dihydroxy-2-(3-hydroxy-4-methoxyphenyl) chroman-4-one	A3	47.74	0.27
Fructus aurantii immaturus	MOL005828	Nobiletin	A4	61.67	0.52
Fructus aurantii immaturus	MOL005849	Didymin	AFI13	38.55	0.24
Fructus aurantii immaturus	MOL000006	Luteolin	AFI14	36.16	0.25
Fructus aurantii immaturus	MOL007879	Tetramethoxyluteolin	AFI15	43.68	0.37
Fructus aurantii immaturus	MOL009053	4-((2S, 3R)-5-((E)-3-hydroxyprop-1-enyl)-7-methoxy-3-methylol-2, 3-dihydrobenzofuran-2-yl)-2-methoxy-phenol	AFI16	50.76	0.39
Licorice	MOL001484	Inermine	GRER1	75.18	0.54
Licorice	MOL001792	DFV	GRER2	32.76	0.18
Llicorice	MOL000211	Mairin	A7	55.38	0.78
Licorice	MOL002311	Glycyrol	GRER3	90.78	0.67
Licorice	MOL000239	Jaranol	GRER4	50.83	0.29
Licorice	MOL002565	Medicarpin	GRER5	49.22	0.34
Licorice	MOL000354	Isorhamnetin	A1	49.6	0.31
Licorice	MOL000359	Sitosterol	C	36.91	0.75
Licorice	MOL003656	Lupiwighteone	GRER6	51.64	0.37
Licorice	MOL003896	7-methoxy-2-methyl isoflavone	GRER7	42.56	0.2
Licorice	MOL000392	Formononetin	GRER8	69.67	0.21
Licorice	MOL000417	Calycosin	GRER9	47.75	0.24
Licorice	MOL000422	Kaempferol	B1	41.88	0.24
Licorice	MOL004328	Naringenin	B2	59.29	0.21
Licorice	MOL004805	(2S)-2-[4-hydroxy-3-(3-methylbut-2-enyl)phenyl]-8, 8-dimethyl-2, 3-dihydropyrano[2, 3-f]chromen-4-one	GRER10	31.79	0.72
Licorice	MOL004808	Glyasperin B	GRER11	65.22	0.44
Licorice	MOL004810	Glyasperin F	GRER12	75.84	0.54
Licorice	MOL004811	Glyasperin C	GRER13	45.56	0.4
Licorice	MOL004814	Isotrifoliol	GRER14	31.94	0.42
Licorice	MOL004815	(E)-1-(2, 4-dihydroxyphenyl)-3-(2, 2-dimethylchromen-6-yl)prop-2-en-1-one	GRER15	39.62	0.35
Licorice	MOL004820	Kanzonols W	GRER16	50.48	0.52
Licorice	MOL004824	(2S)-6-(2, 4-dihydroxyphenyl)-2-(2-hydroxypropan-2-yl)-4-methoxy-2, 3-dihydrofuro(3, 2-g)chromen-7-one	GRER17	60.25	0.63
Licorice	MOL004827	Semilicoisoflavone B	GRER18	48.78	0.55
Licorice	MOL004828	Glepidotin A	GRER19	44.72	0.35
Licorice	MOL004833	Phaseolinisoflavan	GRER20	32.01	0.45
Licorice	MOL004835	Glypallichalcone	GRER21	61.6	0.19
Licorice	MOL004838	8-(6-hydroxy-2-benzofuranyl)-2, 2-dimethyl-5-chromenol	GRER22	58.44	0.38
Licorice	MOL004841	Licochalcone B	GRER23	76.76	0.19
Licorice	MOL004848	Licochalcone G	GRER24	49.25	0.32
Licorice	MOL004849	3-(2, 4-dihydroxyphenyl)-8-(1, 1-dimethylprop-2-enyl)-7-hydroxy-5-methoxy-coumarin	GRER25	59.62	0.43
Licorice	MOL004855	Licoricone	GRER26	63.58	0.47
Licorice	MOL004856	Gancaonin A	GRER27	51.08	0.4
Licorice	MOL004857	Gancaonin B	GRER28	48.79	0.45
Licorice	MOL004860	Licorice glycoside E	GRER29	32.89	0.27
Licorice	MOL004863	3-(3, 4-dihydroxyphenyl)-5, 7-dihydroxy-8-(3-methylbut-2-enyl)chromone	GRER30	66.37	0.41
Licorice	MOL004864	5, 7-dihydroxy-3-(4-methoxyphenyl)-8-(3-methylbut-2-enyl)chromone	GRER31	30.49	0.41
Licorice	MOL004866	2-(3, 4-dihydroxyphenyl)-5, 7-dihydroxy-6-(3-methylbut-2-enyl)chromone	GRER32	44.15	0.41
Licorice	MOL004879	Glycyrin	GRER33	52.61	0.47
Licorice	MOL004882	Licocoumarone	GRER34	33.21	0.36
Licorice	MOL004883	Licoisoflavone	GRER35	41.61	0.42
Licorice	MOL004884	Licoisoflavone B	GRER36	38.93	0.55
Licorice	MOL004885	Licoisoflavanone	GRER37	52.47	0.54
Licorice	MOL004891	Shinpterocarpin	GRER38	80.3	0.73
Licorice	MOL004898	(E)-3-(3, 4-dihydroxy-5-(3-methylbut-2-enyl)phenyl)-1-(2, 4-dihydroxyphenyl)prop-2-en-1-one	GRER39	46.27	0.31
Licorice	MOL004903	Liquiritin	GRER40	65.69	0.74
Licorice	MOL004904	Licopyranocoumarin	GRER41	80.36	0.65
Licorice	MOL004905	3, 22-dihydroxy-11-oxo-delta(12)-oleanene-27-alpha-methoxycarbonyl-29-oic acid	GRER42	34.32	0.55
Licorice	MOL004907	Glyzaglabrin	GRER43	61.07	0.35
Licorice	MOL004908	Glabridin	GRER44	53.25	0.47
Licorice	MOL004910	Glabranin	GRER45	52.9	0.31
Licorice	MOL004911	Glabrene	GRER46	46.27	0.44
Licorice	MOL004912	Glabrone	GRER47	52.51	0.5
Licorice	MOL004913	1, 3-dihydroxy-9-methoxy-6-benzofurano(3, 2-c)chromenone	GRER48	48.14	0.43
Licorice	MOL004914	1, 3-dihydroxy-8, 9-dimethoxy-6-benzofurano(3, 2-c)chromenone	GRER49	62.9	0.53
Licorice	MOL004915	Eurycarpin A	GRER50	43.28	0.37
Licorice	MOL004917	Glycyroside	GRER51	37.25	0.79
Licorice	MOL004924	(-)-medicocarpin	GRER52	40.99	0.95
Licorice	MOL004935	Sigmoidin-B	GRER53	34.88	0.41
Licorice	MOL004941	(2R)-7-hydroxy-2-(4-hydroxyphenyl)chroman-4-one	GRER54	71.12	0.18
Licorice	MOL004945	(2S)-7-hydroxy-2-(4-hydroxyphenyl)-8-(3-methylbut-2-enyl)chroman-4-one	GRER55	36.57	0.32
Licorice	MOL004948	Isoglycyrol	GRER56	44.7	0.84
Licorice	MOL004949	Isolicoflavonol	GRER57	45.17	0.42
Licorice	MOL004959	1-methoxyphaseollidin	GRER58	69.98	0.64
Licorice	MOL004961	Quercetin der.	GRER59	46.45	0.33
Licorice	MOL000497	Licochalcone a	GRER60	40.79	0.29
Licorice	MOL004974	3′-methoxyglabridin	GRER61	46.16	0.57
Licorice	MOL004978	2-((3R)-8, 8-dimethyl-3, 4-dihydro-2H-pyrano(6, 5-f)chromen-3-yl)-5-methoxyphenol	GRER62	36.21	0.52
Licorice	MOL004980	Inflacoumarin A	GRER63	39.71	0.33
Licorice	MOL004985	Icos-5-enoic acid	GRER64	30.7	0.2
Licorice	MOL004988	Kanzonol F	GRER65	32.47	0.89
Licorice	MOL004989	6-prenylated eriodictyol	GRER66	39.22	0.41
Licorice	MOL004990	7, 2′, 4′-trihydroxy–5-methoxy-3–arylcoumarin	GRER67	83.71	0.27
Licorice	MOL004991	7-acetoxy-2-methylisoflavone	GRER68	38.92	0.26
Licorice	MOL004993	8-prenylated eriodictyol	GRER69	53.79	0.4
Licorice	MOL004996	Gadelaidic acid	GRER70	30.7	0.2
Licorice	MOL000500	Vestitol	GRER71	74.66	0.21
Licorice	MOL005000	Gancaonin G	GRER72	60.44	0.39
Licorice	MOL005001	Gancaonin H	GRER73	50.1	0.78
Licorice	MOL005003	Licoagrocarpin	GRER74	58.81	0.58
Licorice	MOL005007	Glyasperins M	GRER75	72.67	0.59
Licorice	MOL005008	Glycyrrhiza flavonol A	GRER76	41.28	0.6
Licorice	MOL005012	Licoagroisoflavone	GRER77	57.28	0.49
Licorice	MOL005013	18*α*-hydroxyglycyrrhetic acid	GRER78	41.16	0.71
Licorice	MOL005016	Odoratin	GRER79	49.95	0.3
Licorice	MOL005017	Phaseol	GRER80	78.77	0.58
Licorice	MOL005018	Xambioona	GRER81	54.85	0.87
Licorice	MOL000098	Quercetin	A2	46.43	0.28
Rhizoma atractylodis macrocephalae	MOL000028	*α*-amyrin	AMR1	39.51	0.76
Rhizoma atractylodis macrocephalae	MOL000033	(3S, 8S, 9S, 10R, 13R, 14S, 17R)-10, 13-dimethyl-17-((2R, 5S)-5-propan-2-yloctan-2-yl)-2, 3, 4, 7, 8, 9, 11, 12, 14, 15, 16, 17-dodecahydro-1H-cyclopenta[a]phenanthren-3-ol	AMR2	36.23	0.78
Rhizoma atractylodis macrocephalae	MOL000072	8*β*-ethoxy atractylenolide III	AMR3	35.95	0.21
Radix saposhnikoviae	MOL011732	Anomalin	SR1	59.65	0.66
Radix saposhnikoviae	MOL011737	Divaricatacid	SR2	87	0.32
Radix saposhnikoviae	MOL011740	Divaricatol	SR3	31.65	0.38
Radix saposhnikoviae	MOL001941	Ammidin	A5	34.55	0.22
Radix saposhnikoviae	MOL011747	Ledebouriellol	SR4	32.05	0.51
Radix saposhnikoviae	MOL002644	Phellopterin	SR5	40.19	0.28
Radix saposhnikoviae	MOL000359	Sitosterol	C	36.91	0.75
Radix saposhnikoviae	MOL000173	Wogonin	SR6	30.68	0.23
Radix saposhnikoviae	MOL000358	Beta-sitosterol	A6	36.91	0.75
Radix saposhnikoviae	MOL001494	Mandenol	SR7	42	0.19
Radix saposhnikoviae	MOL001942	Isoimperatorin	SR8	45.46	0.23
Radix saposhnikoviae	MOL003588	Prangenidin	SR9	36.31	0.22
Radix saposhnikoviae	MOL007514	Methyl icosa-11, 14-dienoate	SR10	39.67	0.23
Radix saposhnikoviae	MOL013077	Decursin	SR11	39.27	0.38
Orange peel	MOL000359	Sitosterol	C	36.91	0.75
Orange peel	MOL004328	Naringenin	B2	59.29	0.21
Orange peel	MOL005100	5, 7-dihydroxy-2-(3-hydroxy-4-methoxyphenyl)chroman-4-one	A3	47.74	0.27
Orange peel	MOL005828	Nobiletin	A4	61.67	0.52

## Data Availability

The data used to support the findings of this study are available from the corresponding author upon request.
